# Characterization of the Correlation Between Ages at Entry Into Breast and Pubic Hair Development

**DOI:** 10.1016/j.annepidem.2010.02.005

**Published:** 2010-05

**Authors:** Krista Yorita Christensen, Mildred Maisonet, Carol Rubin, W. Dana Flanders, Carolyn Drews-Botsch, Celia Dominguez, Michael A. McGeehin, Michele Marcus

**Affiliations:** a Epidemiology Department, Rollins School of Public Health, Emory University, Atlanta, GA; b National Center for Environmental health, Centers for Disease Control and Prevention, Atlanta, GA; c Hawaii Center for Reproductive Medicine, IVF Centers of Excellence, Honolulu, Hawaii

**Keywords:** ALSPAC, Maximum Likelihood Estimation, Puberty, ALSPAC, Avon Longitudinal Study of Parents and Children, BMI, body mass index

## Abstract

**Purpose:**

The timing of breast and pubic hair development in girls are related, but the degree of correlation has not been well characterized. Periodic observations also are complicated by interval censoring.

**Methods:**

Data used were from the Avon Longitudinal Study of Parents and Children. Mean age at entry into breast and pubic hair development was determined by the use of parametric survival analysis. The bivariate normal cumulative distribution function was evaluated over the region containing the paired event times; the likelihood was maximized with respect to the correlation coefficient ρ.

**Results:**

Among 3938 participants, estimated mean ages at entry into Tanner stage 2 for breast and pubic hair development were 10.19 and 10.95, respectively. The likelihood was maximized at ρ = 0.503 to 0.506. This value remained relatively constant among subgroups, although some heterogeneity was observed by maternal and child body mass index and birth order.

**Conclusions:**

The timing of breast and of pubic hair development is moderately correlated and remain so when it is stratified by characteristics associated with puberty.

## Introduction

Although average ages at attainment of female secondary sexual characteristics have been estimated for various populations, the interrelationship of the timing of these events is not well characterized. One reason is that many studies of puberty are cross-sectional in nature, using “status quo” assessments of development, to generate population estimates of timing of various stages. However, individual-level estimates are not possible in a cross-sectional setting. Consequently, if individuals are not followed over time, it is difficult to estimate the correlation between the ages at beginning breast development and pubic hair development because both of these events are not likely to be observed in the same girl. Even in longitudinal studies, the exact timing of events may not be known because the transition generally occurs between study assessments or clinic visits. This phenomenon of interval censored data, where the event is only known to occur within some interval, must be accounted for in estimating age at event.

In the few longitudinal studies that have attempted to estimate the correlation between beginning breast and pubic hair development, two approaches have been used. These are (i) estimate age at event as the age at the first “post-event” study assessment, or (ii) estimate age at event as the midpoint between the last “pre-event” assessment and the first “post-event” assessment. The first method is likely to skew the age distribution toward older ages; the second method is more likely to estimate age at event correctly but also could present problems if there are few assessments or long intervals between assessments.

These methodological considerations may explain some of the variation in estimated correlation between age at beginning breast and pubic hair development among studies. Largo et al. (1) estimated a correlation of 0.34 among girls participating in the First Zurich Longitudinal study, while Nicholson et al. (2) report a correlation of 0.74 among girls participating in the Guidance Study (Table 1) (1–4). Estimates from the Swedish Growth study (3) and the Fels study (4) are intermediate (0.70 and 0.66, respectively). Notably, these studies were reported between 1948 and 1983; there are no estimates from a contemporary longitudinal cohort. Further, it is not known which demographic or physiological characteristics might have an impact on the relationship between timing of breast and pubic hair development.

We used the Avon Longitudinal Study of Parents and Children (ALSPAC) to estimate the correlation between ages at beginning breast and pubic hair development by using a maximum likelihood approach adapted to accommodate interval censored data.

## Materials and Methods

When considering breast and pubic hair development separately, age at occurrence may be treated as a survival time, perhaps following a univariate normal distribution. To characterize the relationship between ages at the two events, a natural extension is the bivariate normal distribution. Parametric survival analyses allow the estimation of the mean (and standard deviation) age at event. However, the correlation between ages at occurrence of the two events is not easily obtained. Most extensions of survival analysis for multiple outcomes use stratification or competing risk scenarios, in which the correlation is either accounted for but not estimated or only one outcome is observed per individual. A further difficulty is introduced by interval censoring of event times. To estimate the correlation between ages at occurrence of two distinct and observed events, a maximum likelihood approach will be used, with modification to accommodate the interval censored nature of the available data.

A pair of event times, defined as *X* for the first event and *Y* for the second, follow some joint probability density distribution *f_X,Y_*(*x*, *y*). The corresponding cumulative distribution function of the paired variables, *F_X,Y_*(*x*, *y*) = *P*(*X*≤*x*, *Y*≤*y*). If *X* and *Y* follow a bivariate normal distribution, *f_X_*_,*Y*_(*x*, *y*) takes the following form (equation 1):

(1) $$f\left(x,y\right)=\frac{1}{2\pi \sigma_{x}\sigma_{y}\sqrt{\left(1-\rho^{2}\right)}}\exp\left[\frac{-1}{2\left(1-\rho^{2}\right)}\left[{\left(\frac{x-\mu_{x}}{\sigma_{x}}\right)}^{2}+{\left(\frac{y-\mu_{y}}{\sigma_{y}}\right)}^{2}-2\rho \left(\frac{x-\mu_{x}}{\sigma_{x}}\right)\left(\frac{y-\mu_{y}}{\sigma_{y}}\right)\right]\right]$$

whereas *F*(*x*, *y*) takes the following form (equation 2):

(2) $$F_{X,Y}\left(x,y\right)=\int_{-\infty }^{x}\int_{-\infty }^{y}f\left(x,t\right)dsdt$$

Because there is no closed expression for *F_X,Y_*(*x*, *y*), the double integrand is approximated with the use of iterative numerical techniques.

In the case of interval censoring, *X* and *Y* are not observed directly, but are known to fall within a certain interval; in the case of left and right censoring, the end point of the interval is negative or positive infinity, respectively. These intervals are indicated as (L_x_, R_x_) for the interval containing x, and (*L_y_*, *R_y_*) for the interval containing *y*. Given these intervals, a region of support can be obtained which contains the pair of event times (*x*, *y*) as follows: [*F_X,Y_*(*R_x_*, *R_y_*) – *F_X,Y_*(*R_x_*, *L_y_*) – *F_X,Y_*(*L_x_*, *R_y_*) + *F_X,Y_*(*L_x_*, *L_y_*)] (5–7). Evaluating *F_X_*_,*Y*_ over this region approximates the likelihood contribution for each individual. To obtain a likelihood function for the cohort, the individual likelihood contributions are multiplied together and the resulting function maximized with respect to ρ. To increase efficiency without loss of specificity, an alternative is to minimize the negative of the natural log of the likelihood.

This method was used to estimate the correlation between age at entry into Tanner stage 2 (or greater, if stage 2 is not observed) of breast and of pubic hair development among girls participating in the ALSPAC (8). The physical development of this cohort has been assessed by the use of annual questionnaires that ascertain Tanner stage of breast and pubic hair development (self-assessed at time of questionnaire completion), mailed to participants from the ages of 8 to 14. Because data were collected annually, entry into breast and pubic hair development is known to occur within intervals defined by timing of questionnaire completion. The median age at entry for each marker and accompanying standard deviation were calculated by the use of the LIFEREG procedure in SAS (SAS Institute, Cary, NC), with interval censoring and the normal distribution specified. This median age is equivalent to the mean, attributable to the normal distribution assumption and the fact that the event occurs within a very specific timeframe during which there are no outliers or skewness. Then, the likelihood procedure was implemented in SAS by use of the PROBBNRM function to estimate the cumulative distribution function of the bivariate normal distribution.

To determine whether the correlation between ages at entry into breast and pubic hair development is a function of some underlying variable, the analysis was repeated for selected maternal and child characteristics that are not intermediate between breast and pubic hair development and are known to be associated with pubertal development. These included mother's prepregnancy body mass index (BMI), birth order, child's BMI at age 8, and child's race. Human subject protection was assessed and approved by the ALSPAC Law and Ethics Committee, the Local Research Ethics Committees, and the Centers for Disease Control and Prevention Institutional Review Board.

## Results

There were 3938 participants with information on breast and pubic hair development. Among these girls, the mean ages at entry into breast stage 2 and pubic hair stage 2 were estimated to be 10.19 (SD 1.52) and 10.95 (SD 1.42). There were 1000 girls who were left censored for age at entry into breast stage 2, and 608 who were left censored for age at entry into pubic hair stage 2; 469 and 712 girls were right censored for age at entry into breast and pubic hair stage 2, respectively. For these girls, the left interval end point was set at (μ – 4*σ) and the right interval endpoint at (μ + 4*σ) as appropriate, where μ and σ represent the estimated mean and standard deviation of age at entry, respectively. The likelihood was evaluated over the range of the correlation coefficient ρ, from −0.999 to 0.999. The negative of the natural log of the likelihood was minimized when ρ reached a value of 0.50.

The correlation between ages at entry into breast and pubic hair stage 2 was similar between subgroups on the basis of maternal and child characteristics associated with pubertal development (Table 2). The greatest difference was found by BMI. The correlation was greatest among girls who were underweight (0.59) or whose mothers were underweight (0.57) and lowest among girls who were overweight or obese (0.31–0.41) or whose mothers were classified as obese (0.41). Correlations ranged from 0.46 to 0.57 when birth order was examined and from 0.50 to 0.53 depending on child's race.

To evaluate the performance of the maximum likelihood approach, the correlation was also estimated by the use of either the end of the interval (i.e., age at completing questionnaire where event is first recorded) or the midpoint of the interval (i.e., age intermediate between questionnaires immediately preceding the event and where event is first recorded). These approaches did not substantially change the estimate of ρ (ρ = 0.56 and 0.50, respectively).

## Discussion

We used data from a contemporary, representative cohort of girls to estimate the correlation between ages at entry into stage 2 of breast and of pubic hair development. By using a novel application of a likelihood maximization technique, we found that approximately one half of the variation in timing of breast development is explained by the variation in timing of pubic hair development, an estimate that is intermediate between (and somewhat lower than most of) previously reported correlations. Reasons for the difference between the present and previously reported studies include use of a different estimation method, different populations and timing, and differences in frequency of study assessments.

It is expected that the timing of breast and pubic hair development will be related because the cascade of events for both is ultimately regulated by the central nervous system. However, the moderate degree of association estimated in this study is consistent with the biology of breast and pubic hair development, which are governed by the hypothalamic pituitary gonadal axis and hypothalamic pituitary adrenal axis, respectively, and thus may not be expected to display a high degree of dependence. Environmental exposures or genetic characteristics that alter production of or response to androgens would be expected to impact timing of pubic hair development, whereas those factors affecting estrogen would have an impact on the timing of breast development. In either case, such disruption could lower the correlation between ages at entry into these developmental milestones.

The magnitude of the association between timing of the two markers was similar within strata of characteristics associated with pubertal development, suggesting that timing of breast and pubic hair development are not independent. That is, differences in the relative timing of breast and pubic hair development do not appear to be caused by the demographic characteristics explored in this analysis. There was some heterogeneity by both child and maternal BMI, however, and the correlation was greatest among underweight girls and girls whose mothers were underweight and lowest among heavier girls and girls whose mothers were heavier. The similarity in pattern by child and maternal BMI may be attributable to the fact that maternal BMI acts as a proxy for girl's BMI (9, 10). Girls who are more overweight may mistake adipose tissue for more advanced breast development, creating spurious differences in timing of maturation for breast and pubic hair development (11, 12).

There were some limitations to these analyses. The correlation represents a linear relationship between variables and thus does not capture more complex dependence that may exist between timing of breast and pubic hair development. Entry into stage 2 was not observed for some girls, only transition from stage 1 to stage ≥3. Because data were collected on an annual basis, events occurring months apart are likely to be reported in the same questionnaire year, which could artificially inflate our estimate of correlation between event times. Finally, correlation is influenced by variance of the individual random variables and covariance between them (13), which may affect subgroup analyses. However, the decreased correlation with greater BMI shows a dose-response relationship not explained by within-strata variance alone.

We considered various distributions in the univariate survival analysis. Mean and median time at entry into breast and pubic hair development were similar under varying distributional assumptions, so the normal distribution was chosen for ease of interpretation. However, this method could be extended to other bivariate distributions, and to higher order distributions, maximizing the likelihood with respect to the covariance matrix. This application of maximum likelihood analysis to interval censored puberty data provides a novel approach for assessing the relationship between timing of milestones in growth and development.

## Figures and Tables

**Table 1:** Estimated correlation coefficients from previous studies

Source	Study	Method	Estimate of ρ
Largo and Prader (1)	First Zurich Longitudinal Study (Switzerland)	Age at observation	0.34
Taranger et al. (3)	Growth study (Sweden)	Midpoint	0.70
Nicholson and Hanley (2)	Guidance Study (CA, USA)	Midpoint	0.74
Reynolds and Wines (4)	Fels Study (OH, USA)	Not stated; probably age at observation	0.66

**Table 2:** Maximum likelihood estimate of the correlation coefficients

Characteristic	n (%)	Correlation
Total	3938 (100)	0.503–0.506
Girl's BMI at 8 years of age		
<5th percentile	69 (2.5)	0.59
5th–85th percentile	2096 (74.7)	0.55–0.56
85th–95th percentile	348 (12.4)	0.31
≥95th percentile	293 (10.4)	0.41
Mother's prepregnancy BMI		
<18.5	186 (5.2)	0.57
18.5–24.9	2671 (75.1)	0.51–0.52
25.0–29.9	516 (14.5)	0.48
≥30.0	183 (5.2)	0.41
Birth order		
First born	1326 (34.6)	0.49
Second born	1277 (33.3)	0.46–0.47
Third born or later	1235 (32.2)	0.57
Child ethnic background		
White	3572 (96.0)	0.50
Nonwhite	148 (4.0)	0.53

BMI = body mass index. Information was missing for some girls, including information on girl's BMI at 8 years of age (n = 1132, 28.8%), mother's prepregnancy BMI (n = 382, 9.7%), girl's birth order (n = 100, 2.5%), and girl's race/ethnicity (n = 218, 5.5%).
